# Supplementary material for: Decoding protein dynamicity in DNA ligase activity through deep learning-based structural ensembles
Source: bioRxiv. 2024 Nov 7:2024.11.07.622521. Preprint. [Version 1] doi: 10.1101/2024.11.07.622521 (PMC11581005; doi:10.1101/2024.11.07.622521)
Supplement: Supplement 1 [file NIHPP2024.11.07.622521v1-supplement-1.pdf]

bioRxiv preprint doi: https://doi.org/10.1101/2024.11.07.622521; this version posted November 7, 2024. The copyright holder for this preprint (which was not certified by peer review) is the author/funder, who has granted bioRxiv a license to display the preprint in perpetuity. It is made available under aCC-BY 4.0 International license.

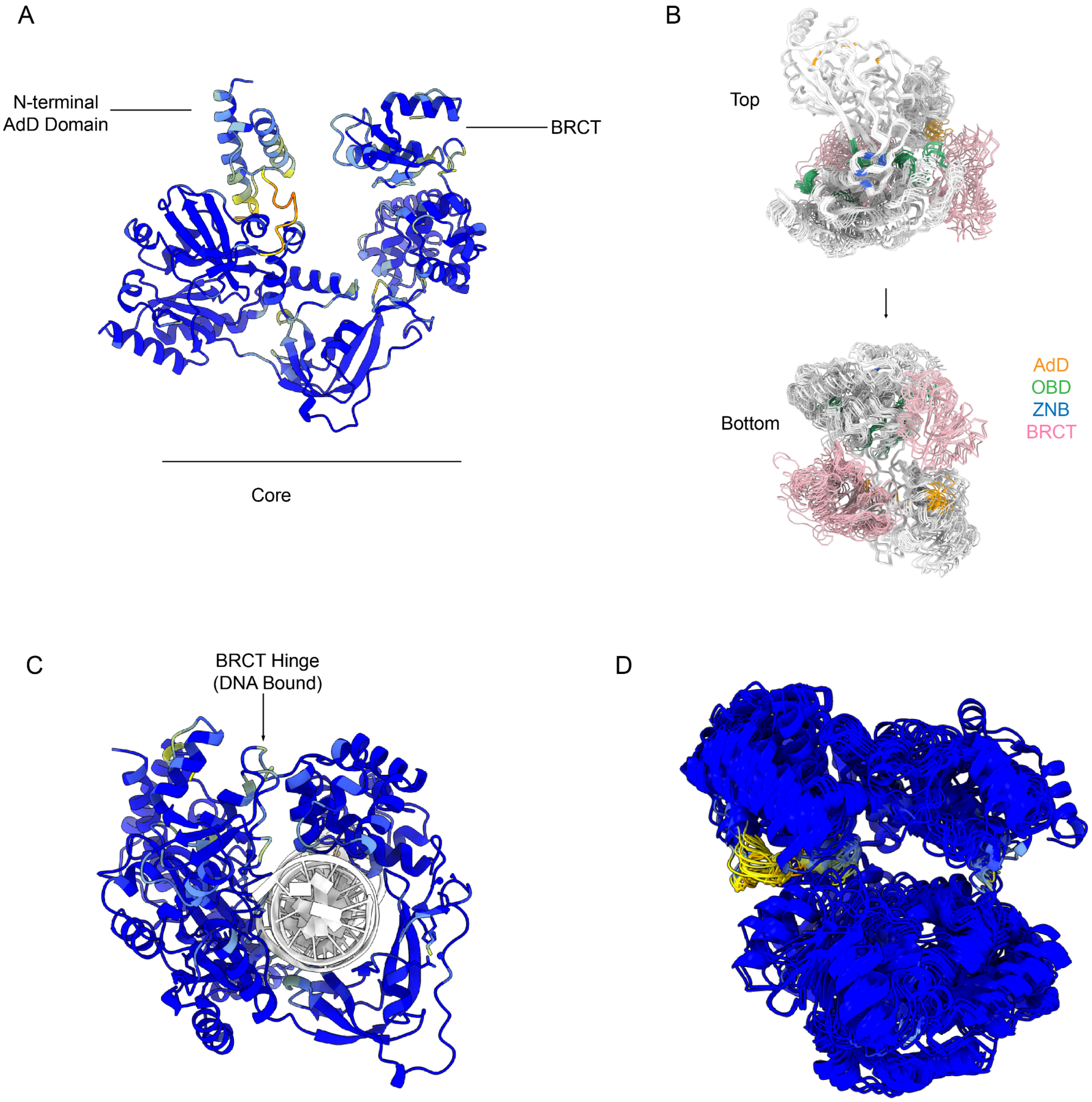

Figure S1: Domain-Specific Confidence and Structural Flexibility in Taq Ligase. (A) Confidence score across domains. (B) Conformational flexibility highlighted by superimposing the ensemble. (C) Hinge confidence in DNA bound form. (D) High confidence in the core domain across the ensemble.
